# Long-Read Sequencing Reveals a GC Pressure during the Evolution of Porcine Endogenous Retrovirus

**DOI:** 10.1128/genomeA.01040-17

**Published:** 2017-10-05

**Authors:** Attila Szűcs, Norbert Moldován, Dóra Tombácz, Zsolt Csabai, Michael Snyder, Zsolt Boldogkői

**Affiliations:** a Department of Medical Biology, Faculty of Medicine, University of Szeged, Szeged, Hungary; b Department of Genetics, School of Medicine, Stanford University, Stanford, California, USA

## Abstract

Here, we present the complete genome sequence of a porcine endogenous retrovirus determined by Pacific Biosciences sequencing. A comparison of the genome of this isolate with those of other strains revealed the operation of a mechanism resulting in the selective accumulation of G and C bases in the viral DNA.

## GENOME ANNOUNCEMENT

Porcine endogenous retrovirus (PERV) is a gammaretrovirus of the *Retroviridae* family (1). It is a provirus inherited in a Mendelian manner, but the pig can also be infected by the virus. PERV is present in multiple copies in the swine genome (1, 2). The virion contains a linear single-stranded (ssRNA)(+) genome.

The PERV genome was reconstructed from the viral transcripts. The viral RNAs were isolated from the porcine kidney cell line PK-15. The cDNA libraries were prepared and sequenced using the Pacific Biosciences RSII platform. Three approaches were carried out for SMRTbell template preparation, nonamplified protocol from poly(A) cDNAs, following the PacBio protocol for very low (10 ng)-input 2-kb libraries with carrier DNA; the amplified Iso-Seq protocol using oligo(dT) primers based on the isoform sequencing (Iso-Seq) protocol with the Clontech SMARTer PCR cDNA synthesis kit; and the modified amplified Iso-Seq protocol using GC-rich random hexamer primers for reverse transcription, as previously described (3). The PacBio DNA template prep kit 1.0 was used for the SMRTbell sequencing template preparation in every case. SMRTbell libraries were bound to polymerases by using the DNA/polymerase binding kit P6 (P/N 100-356-300) and v2 primers. The cDNA sequencing was carried out using the Pacific Biosciences RSII sequencer with P5-C3 reagents for the nonamplified method and P6-C4 reagents for the amplified technique. The movie lengths were 180 min and 240 min, respectively (one movie was recorded for each single-molecule real-time [SMRT] cell).

The sequencing yielded a total of 17,544 reads and a genome coverage of 3,238-fold on average. The average length of the regions of interest (ROIs) was 1,555 nucleotides (nt). Reads were processed and mapped to the genomic sequence with the least variance from our own, that of PERV-60 (GenBank accession no. AY099323), with the Pacific Biosciences SMRT Analysis pipeline (https://github.com/PacificBiosciences/SMRT-Analysis) and GMAP (4).

The genome of PERV strain Szeged is composed of 8,673 bp and contains 3 protein-coding genes.

To pinpoint the genetic events in the adaptation of PERV to the PK-15 cell line, we aligned the genome of PERV strain Szeged to a genome isolated from swine (GenBank accession no. EU789636). Our strain has 50.7% GC content, while its relative isolated from swine has 49.3% GC content. Single-nucleotide variances (a total of 323) between the two genomes show that the rate of AT to GC mutations (179 positions) is 1.62-fold higher than that of GC to AT mutations (110 positions).

When analyzing transcriptome polymorphisms of PERV strain Szeged, we found that the *env* gene harbors the most variable region, with 165 variable nucleotides, while the *gag-pol* gene has 46 variable nucleotides. We also found that the genome has a 1.15 GC/AT ratio. These results also suggest a possible GC pressure on the PERV genome hosted in PK-15 cells.

### Accession number(s).

The complete and annotated genome sequence of PERV subtype A strain Szeged (gag-pol polyprotein and env protein genes) has been deposited in GenBank under accession no. KY484771.
